# Case Report: A Difficult-to-Diagnose Case of Hyperinsulinemic Hypoglycemia Surgically Treated After Developing Acute Pancreatitis

**DOI:** 10.3389/fendo.2021.731071

**Published:** 2021-10-27

**Authors:** Chisa Inoue, Kota Nishihama, Aoi Hayasaki, Yuko Okano, Akinobu Hayashi, Kazuhito Eguchi, Mei Uemura, Toshinari Suzuki, Taro Yasuma, Takeshi Inoue, Tohru Yorifuji, Shugo Mizuno, Esteban C. Gabazza, Yutaka Yano

**Affiliations:** ^1^ Department of Diabetes, Metabolism, and Endocrinology, Mie University Graduate School of Medicine, Tsu, Japan; ^2^ Department of Hepatobiliary Pancreatic and Transplant Surgery, Mie University Graduate School of Medicine, Tsu, Japan; ^3^ Department of Pathology, Mie University Hospital, Tsu, Japan; ^4^ Department of Immunology, Mie University Graduate School of Medicine, Tsu, Japan; ^5^ Department of Pathology, Osaka City General Hospital, Osaka, Japan; ^6^ Division of Pediatric Endocrinology and Metabolism, Children’s Medical Center, Osaka City General Hospital, Osaka, Japan

**Keywords:** congenital hyperinsulinism, insulinoma, diazoxide, hypoglycemia, ABCC8

## Abstract

The patient is a 28-year-old Japanese man diagnosed with severe congenital hyperinsulinemic-hypoglycemia six months after birth. Clinical records revealed no imaging evidence of pancreatic tumor at the time of diagnosis. Subsequently, he had developmental disorders and epilepsy caused by recurrent hypoglycemic attacks. The patient’s hypoglycemia improved with oral diazoxide. However, he developed necrotizing acute pancreatitis at 28 years of age, thought to be due to diazoxide. Discontinuation of diazoxide caused persistent hypoglycemia, requiring continuous glucose supplementation by tube feeding and total parenteral nutrition. A selective arterial secretagogue injection test revealed diffuse pancreatic hypersecretion of insulin. He underwent subtotal distal (72%) pancreatectomy and splenectomy. There was no intraoperative visible pancreatic tumor. His hypoglycemia improved after the surgical procedure. The histopathological study revealed a high density of islets of Langerhans in the pancreatic body and tail. There were large islets of Langerhans and multiple neuroendocrine cell nests in the whole pancreas. Nests of neuroendocrine cells were also detected in lymph nodes. The pathological diagnosis was grade 1 neuroendocrine tumor (microinsulinomas) with lymph node metastases. This patient is a difficult-to-diagnose case of hyperinsulinemic hypoglycemia surgically treated after developing acute pancreatitis. We believe this is a unique case of microinsulinomas with lymph metastases diagnosed and treated as congenital hyperinsulinemic hypoglycemia for almost 28 years.

## Introduction

Endogenous hyperinsulinemic hypoglycemia is generally classified into congenital hyperinsulinemic hypoglycemia (CHI) and acquired hyperinsulinemic hypoglycemia (e.g., insulinoma, insulin autoimmune syndrome, noninsulinoma hypoglycemia syndrome) (1). The diagnosis of CHI is made after excluding causes of acquired hyperinsulinemic hypoglycemia by genetic, biochemical, and imaging studies (1). Although islet cells with large nuclei and cytoplasm are characteristic histopathological findings in CHI (2), pathological findings may partially overlap between CHI and insulinoma (3, 4).

Here we report a case of CHI diagnosed at six months of age that was treated with diazoxide for 28 years. He developed necrotizing acute pancreatitis, thought to be due to diazoxide. The patient underwent subtotal distal pancreatectomy with splenectomy that improved his hypoglycemia. The histopathological examination revealed large and dense areas of islets of Langerhans composed of cells with large and pleomorphic nuclei, suggesting the diagnosis of CHI (2, 5–7). However, nests of pancreatic neuroendocrine tumors were also found in the peripancreatic lymph nodes, suggesting the diagnosis of microinsulinomas with lymph node metastases (8, 9). This paper describes a patient with metastatic microinsulinomas diagnosed with CHI when he was six months old.

## Case Report

**Figure 1:**
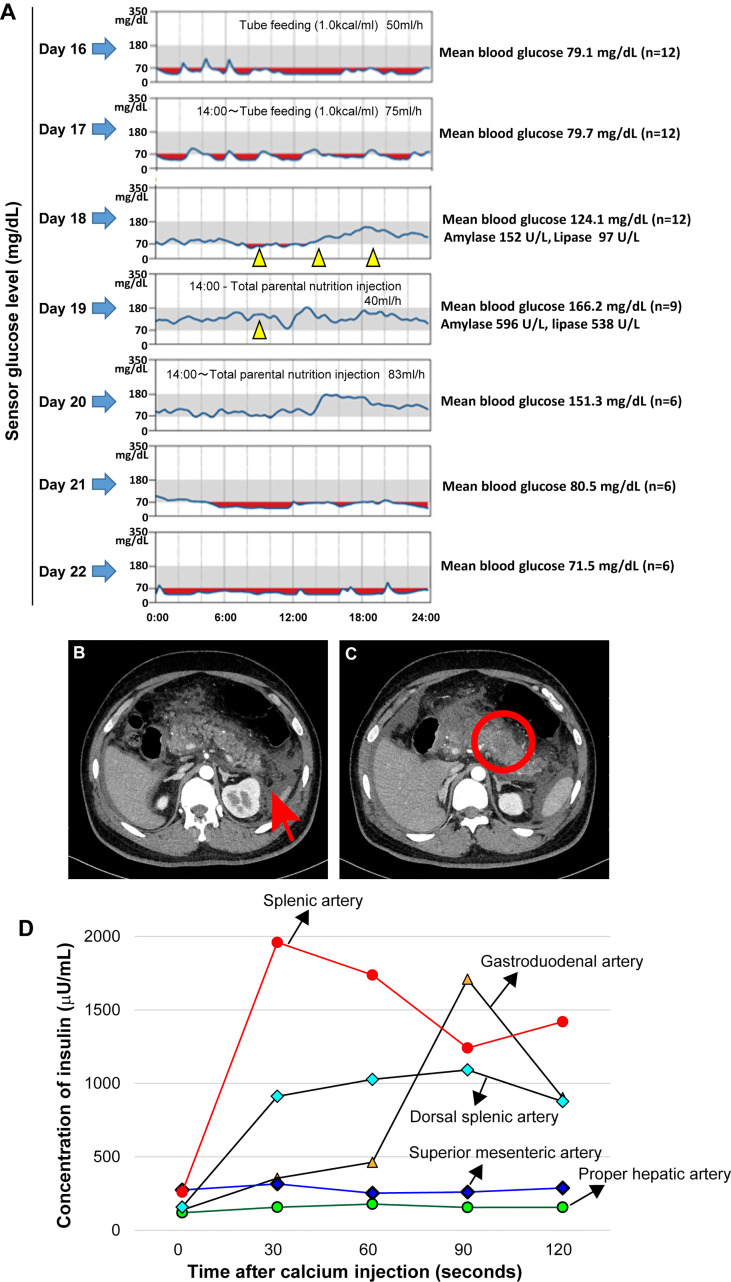
Continuous blood glucose monitoring, mean blood glucose level before and after treatment with diazoxide and enhanced abdominal computer tomography findings compatible with acute necrotizing pancreatitis on admission. Oral diazoxide improved the hypoglycemic state of the patient. The values of amylase and lipase described on day 18 are values before diazoxide treatment. The blood levels of pancreatic amylase (152 →596 U/L) and lipase (97→538 U/L) increased on day 19. Arrow heads indicate treatment with 125 mg diazoxide. Red regions indicate the interval during which the sensor glucose level was less than 70mg/dl. During the tube feeding, we used Peptino (Terumo, Tokyo, Japan), which is a liquid digestive meal. We used ELNEOPA-NF No.2 injection from Otsuka Pharmaceuticals (Tokushima, Japan) for the total parental injection. **(A)**. Enhanced computer tomography (CT) images show enlargement of the pancreas with low-density areas in the pancreatic body **(B)** and peripancreatic fluid collections. There is no evidence of a pancreatic tumor **(C)**. An arterial secretagogue injection (SASI) test shows insulin hypersecretion in the whole pancreases **(D)**. •: Splenic Artery, ▲: Gastroduodenal artery, ◆: Dorsal splenic artery, ◆: Superior mesenteric artery, •: Proper hepatic artery).

A 28-year-old Japanese man consulted Mie University Hospital complaining of fever, abdominal pain, and vomiting. He was born at 38 weeks of gestation with a bodyweight of 3,718 grams. Clinical records revealed no abnormalities in prenatal history, no subtle dysmorphic signs, and no genital-urinary anomalies. Medical history revealed treatment with diazoxide and antiepileptic drugs. There was no imaging evidence of pancreatic tumor at the time of CHI diagnosis. He was diagnosed with CHI at six months of age. There was no history of fetal gigantism, macroglossia, omphalocele, or visceromegaly. In addition, brain magnetic resonance imaging performed when the patient was 23 years old revealed mild atrophy of the cavum septum pellucidum and cerebellum that was considered pathologically not significant. The magnetic resonance imaging revealed no pituitary tumors. During the clinical follow-up, he had developmental disorders, mental retardation, and epilepsy. No further information is available on the cause of brain injury, but we speculate recurrent episodes of severe hypoglycemia caused it. Due to the patient’s significant mental problem, we needed to obtain consent from the parents to perform all studies and treatments. The patient was receiving treatment with diazoxide (375 mg/day), sodium valproate (2,000 mg/day), phenobarbital (210 mg/day) and levetiracetam (3,000 mg/day). The patient was treated with diazoxide until the age of 28 years old. The decrease in episodes of hypoglycemia and laboratory data of glycemia during childhood and monitoring of blood glucose levels (Free-style Libre Pro, Abbott Japan, Tokyo, Japan) after admission to our institution suggest that the patient was responsive to diazoxide (**Figure 1A**). Clinical history also revealed no improvement in the severity of hyperinsulinism in later childhood. He was neither drinker nor smoker. There was no family history of hypoglycemic attacks. The clinical findings on examination at Mie University Hospital were as follows: height 160 cm, body weight 83.4 kg (body mass index 32.5 kg/m^2^), body temperature 39.2 °C, Glasgow Coma Scale E4V3M6, blood pressure 141/102 mmHg, heart rate 158 beats/min, respiratory rate 34/min, peripheral oxygen saturation (SpO_2_) 92% (room air). Physical examination showed a hard distended abdomen with abolished intestinal noises, voluntary muscular defense, and pain on palpation. The laboratory test results, including the elevated serum levels of amylase, were consistent with the diagnosis of acute pancreatitis **(** **Table 1** and **Figure 1A** **)**. There was no sign of primary hyperparathyroidism. An enhanced computer tomography (CT) revealed diffuse pancreas swelling with low-density areas in the pancreas tail, suggesting the presence of necrosis **(** **Figures 1B, C** **)**. CT showed no evidence of pancreatic tumor.

**Table 1:** Laboratory data.

Blood Cell Count & Coagulation			Biochemical Examination		
White blood cell	36,850	/μL	Random Blood Glucose	62	mg/dL
Neutrophil	92.6	%	Total Protein	6	g/dL
Lymphocyte	1.3	%	Albumin	3.3	g/dL
Monocyte	2.8	%	BUN	11.5	mg/dL
Eosinophil	0	%	Creatinine	0.66	mg/dL
Basophil	0	%	eGFR	117.5	mL/min/1.73m^2^
Red blood cell	643	×10^4^/μL	Uric acid	4.4	mg/dL
Hemoglobin	19.7	g/dL	Na	140	mEq/L
Hematocrit	54.2	%	K	4.1	mEq/L
MCV	84.3	fl	Cl	110	mEq/L
MCH	30.6	pg	Ca	7.9	mg/dL
Platelet	19.1	×10^4^/μL	P	1.8	mg/dL
PT	15.6	sec	AST	35	U/L
APTT	38.4	sec	ALT	12	U/L
Fibrinogen	228	mg/dL	LDH	542	U/L
D-dimer	3.13	µg/mL	γ-GTP	43	U/L
			ALP	220	U/L
Arterial Blood Gas Analysis			T-Bil	1.0	mg/dL
pH	7.49		Total Cholesterol	78	mg/dL
pCO_2_	29	mmHg	C-reactive protein	20.29	mg/dL
pO_2_	75	mmHg	Procalcitonin	0.25	ng/mL
HCO_3_^-^	22.1	mmol/L	Amylase	1,033	U/L
Base Excess	0.5	mmol/L			
Lactic acid	3	mmol/L	Auto-antibody		
			Anti-insulin antibody	<0.4	U/mL
Results of laboratory data during hypoglycemia*					
Blood Glucose	59	mg/dL			
Insulin	20.6	μIU/mL	Octreotide test **		
Serum C-peptide	4.2	ng/mL	Time (hr)	Blood Glucose (mg/dL)	
Total ketone body	220	μmol/L	0	56	
Acetoacetic acid	114	μmol/L	1	67	
3-hydroxybutyric acid	106	μmol/L	2	78	
Cortisol	19.7	μg/dL	3	67	
Adrenocorticotropic hormone	24	pg/mL	4	70	
Growth hormone	1.15	ng/mL	6	91	
IGF-1	87	ng/mL	8	48	
Adrenaline	97	pg/mL	12	66	
Noradrenaline	144	pg/mL			
Dopamine	8	pg/mL			

*Performed on Day 11; **Performed on Day 194.

Intravenous rehydration and antibiotics were indicated. Because of the possibility of drug-induced pancreatitis, diazoxide and antiepileptic drugs were discontinued. However, after discontinuation of diazoxide, the patient had refractory hypoglycemia caused by hyperinsulinemia **(** **Table 1** and **Figure 1A** **).** After obtaining informed consent from the parents, 375 mg/day of diazoxide was resumed on day 18. However, the blood levels of pancreatic amylase (152→596 U/L) and lipase (97→538 U/L) further increased on day 19 (**Figure 1A**). Therefore, diazoxide was considered the causative agent of pancreatitis and was discontinued on day 19. The control of glucose with a combination of diet therapy, tube feeding, and total parenteral nutrition (TPN) was difficult after discontinuing diazoxide. An octreotide test (100 μg, subcutaneous) showed that hypoglycemia would be difficult to control using somatostatin receptor ligands **(** **Table 1** **).** The diagnosis of the pancreatic tumor using imaging studies was difficult due to acute pancreatitis and Walled-off necrosis. We performed a selective arterial secretagogue injection (SASI) test to assess whether insulin hypersecretion was in the whole pancreas. The SASI test revealed hypersecretion of insulin from the whole pancreas **(** **Figure 1D** **)**. During hospitalization, the patient was also treated for repeated catheter-related bloodstream infection caused by methicillin-resistant *Staphylococcus aureus* and *Candida parapsilosis*. The patient’s general condition improved during his hospitalization for 8 months. However, we indicated subtotal distal pancreatectomy with splenectomy on day 250 when the patient’s general condition deteriorated due to Walled-off necrosis secondary to acute pancreatitis. There was no intraoperative visible pancreatic tumor. The spleen was removed because of adhesiveness to surrounding pancreatic tissue, but it was not pathologically evaluated. The blood glucose levels normalized immediately after pancreatectomy. However, due to subtotal pancreatectomy, the patient developed diabetes mellitus at the early stages of the postoperative period. The patient was discharged on day 290 after admission, and he is now under insulin therapy.

**Figure 2:**
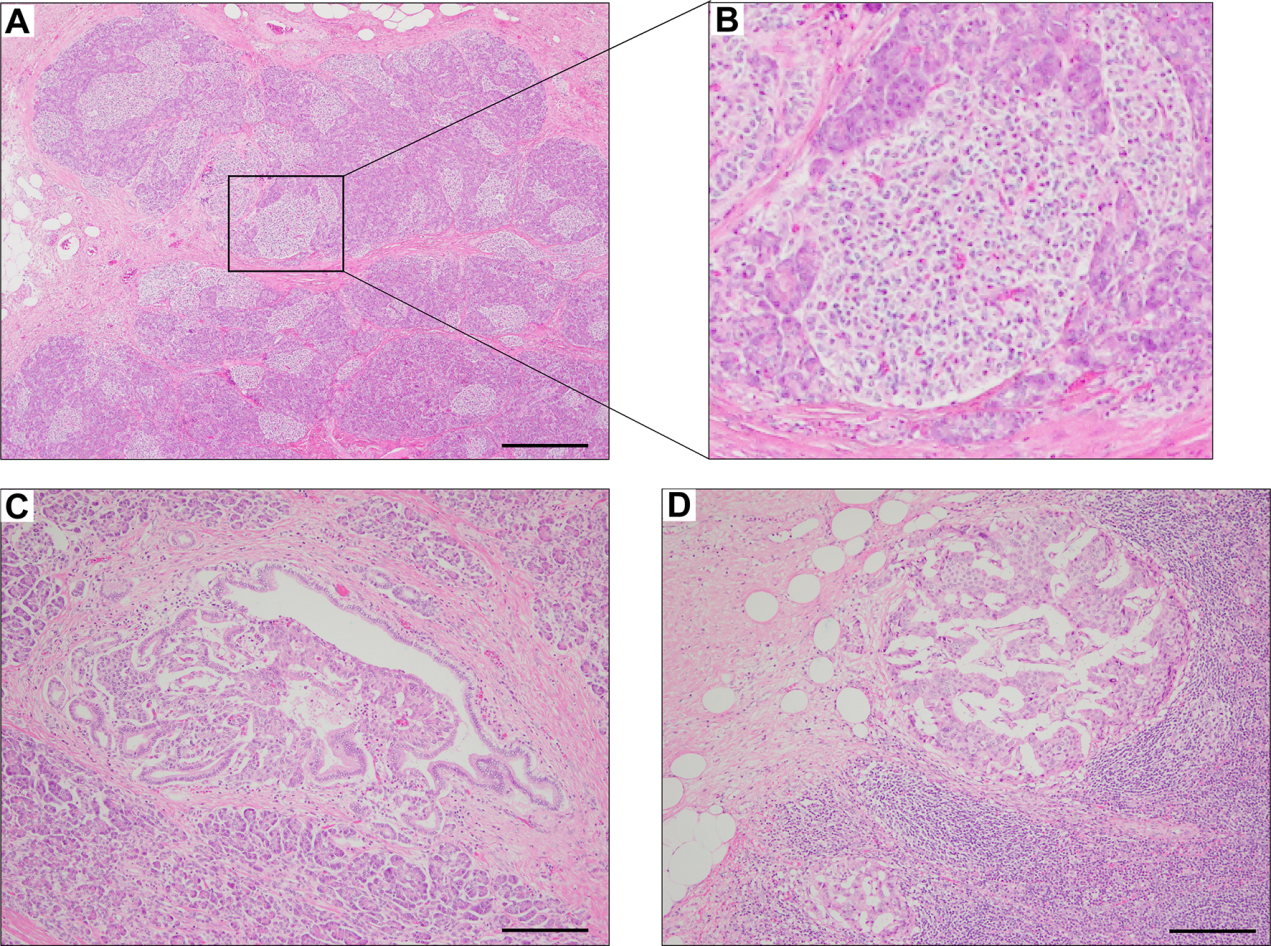
Hematoxylin & eosin staining of pancreas surgical specimen. Staining shows the increased density of Langerhans cells from the body through the tail of the pancreas **(A, B)**, ductuloinsular complex **(C)**, and lymph node metastases **(D)**. Scale bars indicate 500 µm in **(A)** and 200 µm in **(C**, **D)**.

**Figure 3:**
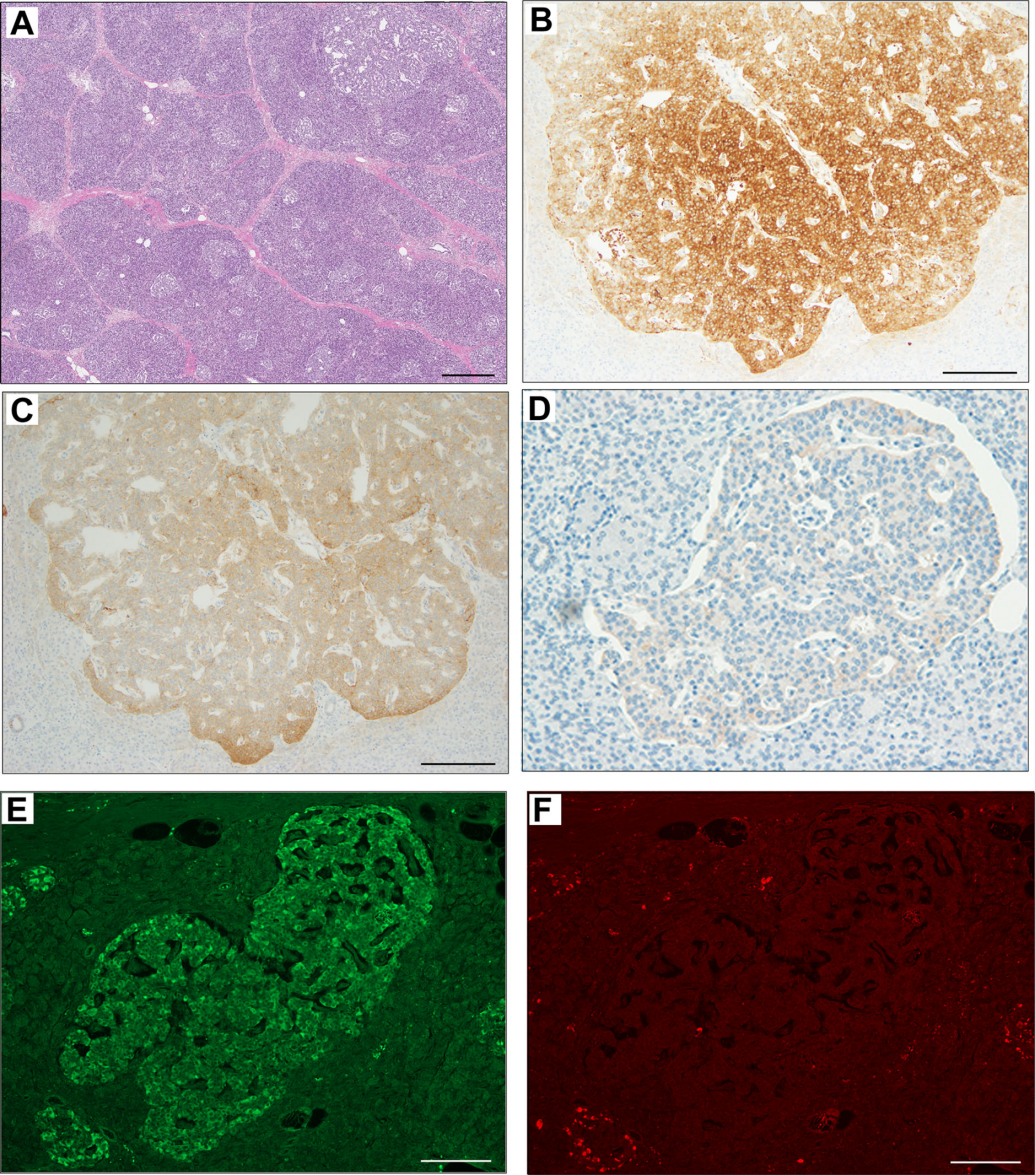
Immunohistochemical staining of pancreas surgical specimen. Low magnification of pancreatic tail tissue stained with hematoxylin & eosin **(A)**. Immunohistochemical staining of pancreatic tail tissue showed positivity for synaptophysin **(B)**, neural cell adhesion molecule or CD56 **(C)**, somatostatin receptor 2 **(D)**, and insulin **(E)**. Glucagon staining was negative **(F)**. Scale bar indicates 500 µm in **(A)** and 200 µm in **(B–F)**.

The pathological findings of the surgical samples were independently evaluated by expert pathologists (Dr. Akinobu Hayashi from Mie University Hospital and Dr. Takeshi Inoue from Osaka City General Hospital) from different institutions. In addition, the pathological study showed large-sized islets of Langerhans distributed in the whole pancreas, with the density of islets increasing from the body through the tail of the pancreas (**Figures 2A, B**). Importantly, in addition to the ductuloinsular complex in the pancreas (**Figure 2C**), the histopathological examination of peripancreatic lymph nodes showed the presence of nests of neuroendocrine cells (**Figure 2D** and **Supplementary Figures 1A–C**). Additional pathological findings were neuroendocrine cell nests larger than 1 mm with pleomorphic and enlarged nuclei, positive staining for synaptophysin and neural cell adhesion molecule (CD56), and positive staining for somatostatin receptor 2 (SSTR2a) in the pancreatic tail (**Figures 3A–D**). Immunofluorescence staining also demonstrated an increased number of insulin-producing β-cells in the patient’s pancreas (**Figures 3E, F**). The diagnosis of the pathologists was a G1 neuroendocrine tumor (microinsulinomas) with lymph node metastases. The MIB-1 (Ki-67) labeling index was 1.6%. Genetic analyses of leukocyte genomic DNA from the patient and his parents were performed using the BigDye™ Terminator v3.1 Cycle Sequencing Kit (ThermoFisher, Waltham, MA) and the ABI Prism 3130xl Sequencer (Applied Biosystems, Waltham, MA). The genetic study in the patient and his father revealed a genetic variant (heterozygous adenosine triphosphate (ATP)-binding cassette subfamily c member 8 [*ABCC8*] intron33 c.4120-19C>T) in the ATP-sensitive potassium channel (K_ATP_) gene. His mother showed no genetic abnormalities. Leukocyte genomic DNA was also used to test for MAFA. Missense mutation of MAFA (v-maf musculoaponeurotic fibrosarcoma oncogene homolog A) gene, a cause of familial insulinoma, was not detected (10). A congenital hyperinsulinemia gene panel test performed using pancreatic tissue genomic DNA also revealed the same heterozygous ABCC8 variant as detected in blood. In the gene panel test using pancreatic tissue genomic DNA, no pathogenic mutation was detected in other genes that may cause hypoglycemia, including HNF1A, HNF4A, KCNJ11, GCK, HADH, UCP2, GLUD1, SLC16A1, IR, HK1, PGM1, PMM2, FOXA2, CACNA1D, MPI, ALG3, KDM6A, KMT2A, NSD1, DIS3L2, HRAS, CACNA1C, and GPC3 genes (3, 11, 12). We performed methylation-specific multiplex ligation-dependent probe amplification (MS-MLPA, ME030-C3, MRC-Holland, Amsterdam, The Netherlands).using blood samples and found no genetic abnormalities compatible with BWS.

## Discussion

CHI is a group of genetic disorders that cause persistent hypoglycemia (11, 12). CHI is histopathologically classified in focal, diffuse, and atypical (morphological mosaicism) types (2, 3, 7, 11, 13). Pancreatic islet cells with large nuclei and cytoplasm are present in any form of CHI (2, 3, 11). CHI is diagnosed by the history of clinical episodes and laboratory tests consistent with hyperinsulinemic hypoglycemia after excluding causes of acquired hyperinsulinemia (1). Several gene mutations can cause CHI (12). Therefore the genetic analysis is now an indispensable tool for CHI diagnosis (1). Hypoglycemia was relatively well controlled in our present case with oral diazoxide for almost 28 years. Long-term administration of diazoxide for up to 7 years in patients with hyperinsulinemic hypoglycemia and 27 years in patients with insulinoma has been previously reported (14–16). Unfortunately, his hypoglycemia became uncontrollable when he developed acute pancreatitis with Walled-off necrosis thought to be due to diazoxide at 28 years of age. Imaging studies failed to show tumors consistent with the diagnosis of insulinoma. Subcutaneous administration of octreotide, tube feeding, and total parenteral nutrition was indicated to treat his hypoglycemia after diazoxide discontinuation. However, these treatments were ineffective. Although the surgical approach of Walled-off necrosis secondary to acute pancreatitis remains controversial (17, 18), we considered the surgical approach because some studies reported the efficacy of pancreatectomy in CHI (1, 19). Subtotal distal pancreatectomy with splenectomy was finally indicated after a selective arterial secretagogue injection (SASI) test confirmed diffuse pancreatic hypersecretion of insulin. We observed no visible pancreatic tumor during the operation. Hypoglycemia ameliorated after the surgical procedure.

The pathological examination of the surgical specimens from this patient disclosed unique findings. Histological examination revealed large islets of Langerhans containing large cells with pleomorphic nuclei and focal areas with increased density of islets, suggesting a diffuse form of CHI (7, 13). Interestingly, areas with ductuloinsular complex, which are characteristic findings of the focal form of CHI, were also observed (5). In addition, despite the absence of pancreatic nodules, there were nests of synaptophysin-positive and insulin-positive neuroendocrine cells with pleomorphic nuclei consistent with the diagnosis of microinsulinomas (4, 8, 9). Together with the presence of a metastatic lesions in peripancreatic lymph nodes, these findings support the diagnosis of microinsulinomas with lymph node metastases (International Union Against Cancer Classification: pT1, pN1) (20). Kwon et al. previously reported metastasis in peripancreatic lymph nodes in a patient with a 0.4 cm pancreatic neuroendocrine microadenoma (21). However, to the best of our knowledge, there is no report of lymph node metastases in a patient with an unidentified nodule of insulinoma. Therefore, we believe this is a unique case of microinsulinomas with lymph node metastases without detectable pancreatic nodules. Another unique feature of the current case is the long time delay (28 years) to define the pathological entity of the disease. However, it is worth noting that based on the available clinical, laboratory, and pathological data, it is difficult to assert whether this patient represents a case of microinsulinomas with slow progression, a case of CHI associated with microinsulinomas, or a case of CHI that progressed to microinsulinomas.

The K_ATP_ channel plays a critical role in insulin secretion from pancreatic β-cells (22). Increased glucose metabolism by β-cells in the presence of high intracellular glucose levels accelerates ATP production, leading to the closure of the K_ATP_ channels (23). The closure of K_ATP_ channels prevents the outward flux of potassium ions, which depolarizes the cell membrane (23). Depolarization of the cell membrane activates the voltage-dependent calcium channels leading to increased intracellular calcium levels, which ultimately triggers calcium-dependent exocytosis of insulin (23). The K_ATP_ channels of pancreatic β-cells are composed of a sulfonylurea receptor (SUR1) unit and an inward rectifying potassium channel (Kir6.2) unit (22). The *ABCC8* gene encodes the pancreatic β-cell-expressed SUR1 unit of the K_ATP_ channels (23). A genomic DNA analysis in the present case revealed a mutation in the SUR1 unit (heterozygous *ABCC8* intron33 c.4120-19C>T) of the K_ATP_ channel. Genetic variants of *ABCC8* have been associated with resistance to diazoxide therapy (11). However, according to a recent publication by Flanagan et al. (24) and the Genome Aggregation Database (https://gnomad.broadinstitute.org/variant/11-17417496-G-A?dataset=gnomad_r2_1) the ABCC8 variant detected in our present case is not probably pathogenic. Nonetheless, the significance of this heterozygous ABCC8 variant in insulinoma should be the subject of future studies.

CHI associated with the Beckwith-Wiedemann syndrome (BWS) is a differential diagnosis that needs consideration in the present case. BWS is most frequently caused by a mosaic paternal uniparental disomy in the 11p15.5 gene cluster; that is, the patient has two paternally derived chromosome 11p15 copies with no maternal contribution (25). Apart from hyperinsulinemic hypoglycemia, BWS patients may show somatic overgrowth, postnatal gigantism, visceromegaly, macroglossia, omphalocele, and a high risk of developing benign or malignant neoplasms (26). Also, hyperinsulinism in BWS due specifically to 11pUPD is usually not diazoxide-responsive (27). It is worth noting here that not all hypoglycemia in BWS is resistant to diazoxide treatment, as most cases are not affected by severe hypoglycemia requiring pancreatectomy (26, 28). A methylation-specific multiplex ligation-dependent probe amplification analysis showed negative findings for BWS. However, BWS is mosaic and therefore the test may not have detected abnormal methylation if the tissue tested did not have a high degree of the BWS change. In connection to abnormalities of chromosome 11, multiple endocrine neoplasia type 1 (MEN1), an autosomal dominant disorder, is another differential diagnosis that needs to be considered. MEN1 is characterized by the presence of primary hyperparathyroidism, pituitary tumors, and pancreaticoduodenal neuroendocrine tumors (29). MEN1 mutations and chromosome 11 isodisomy have been involved in a large population of children and adolescents with insulinoma (30). However, based on the lack of characteristic clinical findings or family history of MEN1, lack of clinical and genetic findings of BWS, the clinical course of the disease, the initial responsiveness to dioxide treatment, and the expert pathologists’ final reports, we believe that our patient represents a rare case of microinsulinomas with lymph node metastases.

Diazoxide, a K_ATP_ channel opener, is the first-line therapy for CHI (1). Hypoglycemia in this patient was well controlled with diazoxide therapy for a long time. However, we discontinued diazoxide based on clinical suspicion of drug-induced pancreatitis. Subsequent resumption of oral diazoxide was associated with high levels of pancreatic enzymes that further increased the suspicion of diazoxide-induced pancreatitis. Although the patient received several drugs during the clinical follow-up, serum amylase levels over 500 U/L were detected only between days 19 and 31 after re-starting treatment with oral diazoxide. Moreover, while the CT scan performed on day 19 showed no evidence of acute pancreatitis, the CT scan performed on day 29 showed findings compatible with acute pancreatitis that substantially improved in the CT scan performed 7 days later. In addition, the radiological study showed no other possible causes of pancreatitis, such as stones or mass, and there were no clinical records of hypertriglyceridemia or pancreatitis-associated genetic abnormalities. On these bases, we made the diagnosis of diazoxide-induced acute pancreatitis. De Broe et al. were the first to suggest acute pancreatitis as a potential adverse effect of diazoxide, and Badalov et al. described diazoxide as a rare cause of acute pancreatitis (31, 32). Interestingly, de Oliveira Andrade et al. reported that diazoxide reduces necrosis of acinar cells in rats with acute pancreatitis, although the mortality rate was higher in the diazoxide-treated group than in the untreated group (33). Overall, these observations highlight the importance of considering diazoxide as a potential cause of drug-induced acute pancreatitis in patients receiving the drug for a long term.

## Conclusion

This patient is a difficult-to-diagnose case of hyperinsulinemic hypoglycemia surgically treated after developing diazoxide-related acute pancreatitis. We believe this is a unique case of microinsulinomas with lymph metastases histopathologically showing large islets of Langerhans in the whole pancreas in a patient diagnosed with congenital hyperinsulinemic hypoglycemia for almost 28 years.

## Data Availability Statement

Patient data and the clinical course were retrieved from electronic medical records. They are available from the corresponding author upon reasonable request.

## Ethics Statement

Written informed consent was obtained from the patient’s parents for the publication of clinical details and images. A genetic test was performed following the ethical code of Osaka City General Hospital (approval number 743).

## Author Contributions

CI and KN were equally responsible for clinical treatment, follow-up, and preparation of the manuscript. AoH, YO, KE, MU, and TS were responsible for clinical treatment, follow-up, and interpretation. AkH and TaY were responsible for pathological investigation. TI and ToY were responsible for genetic diagnosis. SM, EG, and YY were responsible for interpreting the data and intellectual contribution in preparing the manuscript. All authors contributed to the article and approved the submitted version.

## Conflict of Interest

YY reports receiving lecture fees from Novo Nordisk.

The remaining authors declare that the research was conducted in the absence of any commercial or financial relationships that could be construed as a potential conflict of interest.

## Publisher’s Note

All claims expressed in this article are solely those of the authors and do not necessarily represent those of their affiliated organizations, or those of the publisher, the editors and the reviewers. Any product that may be evaluated in this article, or claim that may be made by its manufacturer, is not guaranteed or endorsed by the publisher.
