# Supplementary material for: Case Report: A Difficult-to-Diagnose Case of Hyperinsulinemic Hypoglycemia Surgically Treated After Developing Acute Pancreatitis
Source: Front Endocrinol (Lausanne). 2021 Oct 27;12:731071. doi: 10.3389/fendo.2021.731071 (PMC8578890; doi:10.3389/fendo.2021.731071)
Supplement: Supplementary file 1 [file DataSheet_1.docx]

**A difficult-to-diagnose case of hyperinsulinemic hypoglycemia surgically treated after developing acute pancreatitis**

Chisa Inoue, Kota Nishihama, Aoi Hayasaki, Yuko Okano, Akinobu Hayashi,

Kazuhito Eguchi, Mei Uemura, Toshinari Suzuki, Taro Yasuma, Takeshi Inoue,

Tohru Yorifuji, Shugo Mizuno, Esteban C. Gabazza, Yutaka Yano.


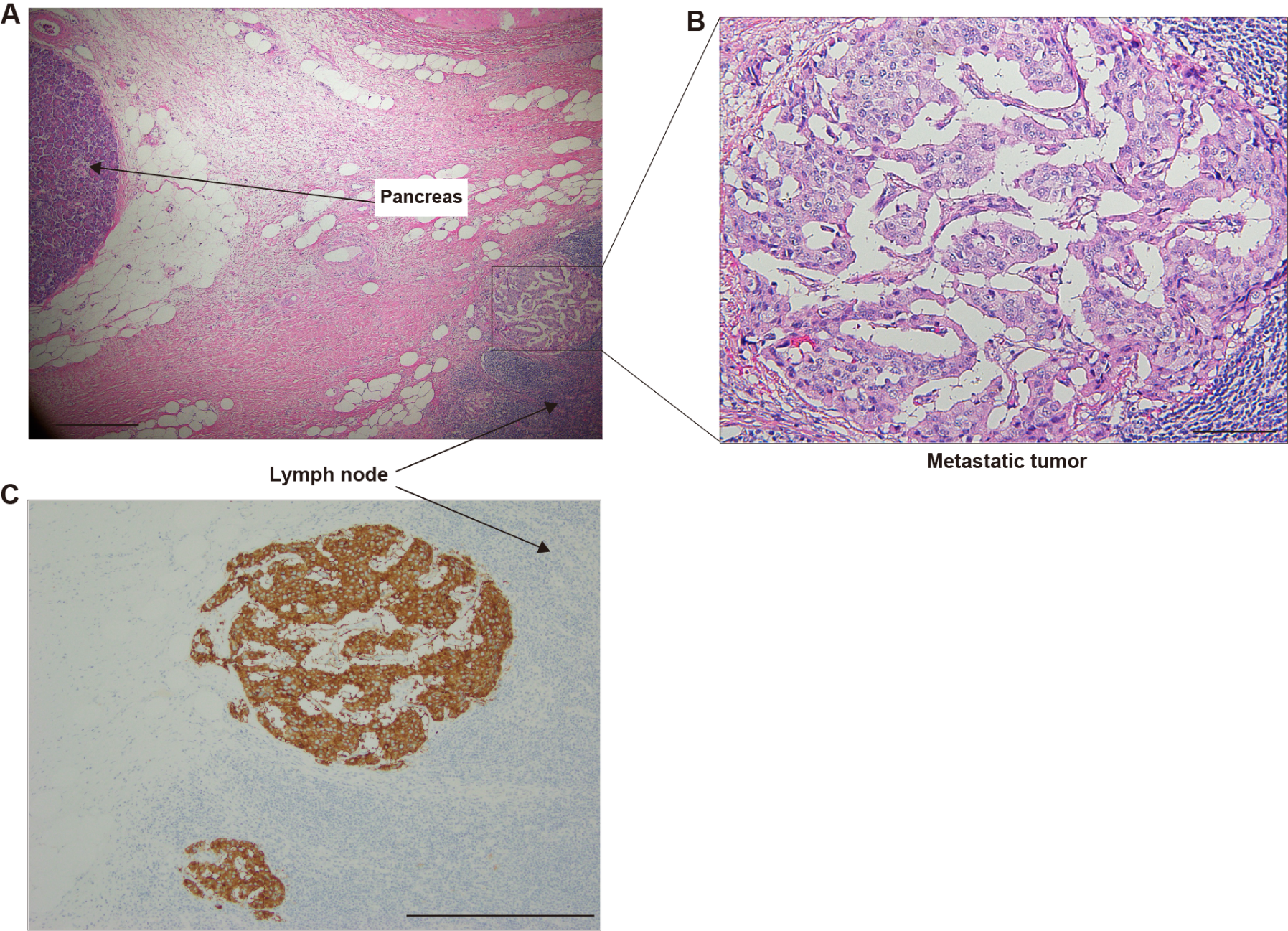


**Supplementary Fig. 1. Peripancreatic region showing a lymph node with metastatic tumor.** The surgical specimens were stained with hematoxylin & eosin and observed under a light microscope. The slide shows the pancreatic tissue (**A**) and a lymph node (**A**, **B**) in the peripancreatic region with a metastatic tumor. Positive staining for synaptophysin (**C**). Scale bars indicate 500 µm in **A** and 100 µm in **B**.
